# Optimization, characterization, and follicular targeting assessment of tretinoin and bicalutamide loaded niosomes

**DOI:** 10.1038/s41598-023-47302-6

**Published:** 2023-11-16

**Authors:** Parisa Ghasemiyeh, Fatemeh Moradishooli, Saeid Daneshamouz, Reza Heidari, Uranous Niroumand, Soliman Mohammadi-Samani

**Affiliations:** 1 Pharmaceutical Sciences Research Center, Shiraz University of Medical Sciences, Shiraz, Iran; 2 Department of Clinical Pharmacy, School of Pharmacy, Shiraz University of Medical Sciences, Shiraz, Iran; 3 Department of Pharmaceutics, School of Pharmacy, Shiraz University of Medical Sciences, Shiraz-Marvdasht Hwy, Karafarin St, Shiraz, Fars Province 71468 64685 Iran; 4 Department of Pharmaceutical Nanotechnology, School of Pharmacy, Shiraz University of Medical Sciences, Shiraz, Iran

**Keywords:** Acne vulgaris, Nanomedicine, Nanoscale materials

## Abstract

Acne vulgaris, a prevalent skin disorder among teenagers and young adults, can have numerous psychological consequences. Topical treatment of acne would be advantageous by reducing the risk of systemic adverse drug reactions. However, the major challenge would be skin penetration through the stratum corneum. Therefore, during this study, tretinoin (TRT) and bicalutamide (BCT) loaded niosomes with follicular targeting potential were fabricated through the thin film hydration technique. Formulation optimization was performed using the Design-Expert software and optimum formulation was characterized in terms of particle size, zeta potential, transmission electron microscopy, drug loading, and differential scanning calorimetry. In vivo follicular targeting was assessed using rhodamine B-loaded niosomes to follow the skin penetration pathways. The results showed that, the optimum formulation was spherical in shape and had an average diameter of 319.20 ± 18.50 nm and a zeta potential of − 29.70 ± 0.36 mV. Furthermore, entrapment efficiencies were 94.63 ± 0.50% and > 99% and loading capacities were 1.40 ± 0.01% and 1.48 ± 0.00% for BCT and TRT, respectively. According to the animal study results, the prepared niosomes with an average diameter of about 300 nm showed significant accumulation in hair follicles. It seems that the designed niosomal BCT-TRT co-delivery system would be promising in acne management with follicular targeting potential.

## Introduction

Acne vulgaris is one of the most prevalent skin disorders worldwide that mainly affects teenagers and young adults^1^. Various pathophysiologic factors can induce or exacerbate acne vulgaris occurrence including androgen-induced enhanced sebum production through the conversion of testosterone to dihydrotestosterone (DHT), bacterial colonization especially *Propionibacterium acnes*, *Malassezia furfur*, and *Staphylococcus epidermidis*, inflammation through the follicle rupture and release of pro-inflammatory chemicals^2^, and hyperproliferation and hyperkeratinization of the hair follicles and pilosebaceous units^3,4^. Acne vulgaris mainly involves the face and trunk areas. It has been reported that about 9.4% of the global population is affected by acne vulgaris and acne has been considered as the eight most prevalent diseases in the world. Based on recent epidemiologic and systematic review studies, acne prevalence has been estimated to be between 20 and 95% with a rising incidence during the late adolescent period between 15 and 19 years old^5^. In addition, it has been reported that 85% of teenagers and young adults (12–24 years old) and 50% of young adults between the ages of 20 and 29 have experienced acne vulgaris^6^. Since acne vulgaris can be accompanied by various psychological consequences including social isolation, low self-esteem, self-harm, suicidal ideation, anxiety, and depression, early management of acne vulgaris, especially in teenagers and young adults, is crucial to prevent these unwanted psychological adverse effects^7^.

Various therapeutic agents including systemic and topical formulations have been considered in acne management based on disease severity. Oral antibiotics, oral retinoids (isotretinoin), and hormonal agents (oral contraceptive agents (OCPs) and anti-androgenic agents) are among the most commonly prescribed systemic therapeutic agents. In addition, topical antibiotics, benzoyl peroxide, topical retinoids, azelaic acid, and salicylic acid are commonly used topical therapeutic options^8^. Although some of these systemic therapeutic agents are significantly effective in acne management, due to various adverse drug reactions, they may be intolerable for some patients^9^. Therefore, the recruitment of topical therapeutic agents with adequate clinical efficacy and much lower adverse drug reactions would be promising. The major challenge of topical treatment of acne lesions would be limited skin penetration through the skin layers especially the stratum corneum layer^10^. In this regard, the use of novel topical drug delivery systems including nanoemulsions, solid lipid nanoparticles (SLNs), nanostructured lipid carriers (NLCs), liposomes, niosomes, polymeric nanoparticles, and nanocrystals have been developed and studied to enhance skin penetration and also to induce targeted drug delivery to skin organelles especially the hair follicles^10,11^. Follicular targeting is a promising approach in acne treatment, as it can enhance dermal bioavailability, increase clinical effectiveness, and reduce systemic adverse drug reactions^12^. Targeted drug delivery to the hair follicles can bypass the stratum corneum as the main challenge in topical drug delivery. Various physicochemical factors of the loaded drug and also the carrier can affect targeted follicular delivery, and particle size plays a crucial role among these factors^13^. Niosomes have shown promising results for targeted follicular delivery, as they can significantly enhance drug accumulation and prolong deposition within the hair follicles^14,15^. Therefore, in this study, niosomes were fabricated, optimized, and characterized for follicular targeting purposes.

Tretinoin (TRT) is a topical retinoid agent that is commercially available in 0.025%, 0.05%, and 0.1% cream, liquid, and gel formulations and has been recommended by the American Academy of Dermatology (AAD) and United States Food and Drug Administration (US FDA) for acne treatment. TRT is a lipophilic agent with PK_a_ of 4.67 and log P of 6.3 which is practically insoluble in water (2.48 × 10^–2^ mg/L). Tretinoin could be easily oxidized and it is a light-sensitive agent^16^. The anti-acne effect of TRT can be attributed to the reduction in keratinocyte proliferation, normalization of follicular differentiation which can lead to pilosebaceous unit unclogging. In addition, TRT exhibits anti-inflammatory properties through the inhibition of bacterial-induced cytokine release and pro-inflammatory pathways^17^. Because topical administration of TRT is challenging due to its photosensitivity, instability, limited water solubility, and skin irritation, TRT-loaded niosomes have been designed and characterized for topical drug delivery purposes with the advantages of reduced skin irritation, enhanced photo-stability, improved skin penetration, and prolonged cutaneous deposition^18–20^.

Bicalutamide (BCT) is a non-steroidal anti-androgenic agent^21^ which is a lipophilic drug with a log P of 2.3 and water solubility of 11.75 mg/L and a pK_a_ value of about 12^13^. Anti-androgenic agents are a class of drugs that can be used in acne treatment, especially in those with hormonal imbalance-induced acne vulgaris. Anti-androgenic agents, including BCT, can suppress or reduce androgen-induced sebum production, making them potentially beneficial in the management of acne^22^. In this regard, co-delivery of BCT and TRT, with at least two different mechanisms of action, to the hair follicles would be promising synergistic compounds in acne management. Niosomes, as non-ionic surfactant-based vesicular nanocarriers, are favorable for trans-follicular drug delivery. Because the niosomes have the potential to merge with skin cell membrane, skin penetration enhancement of the incorporated drug through the stratum corneum layer would be predictable^23^. Therefore, the main goal of this study was to design and develop a novel dual-drug delivery system, BCT-TRT loaded niosomes, for targeted delivery to the hair follicles and pilosebaceous units.

## Results

### Quantitative analysis

Results of RP–HPLC–PDA method validation revealed that this technique could successfully analyze BCT and TRT simultaneously using a photodiode array (PDA) detector at two distinct λ_max_ of 272 and 350 nm, respectively, during a run time of 25 min. This validated technique had sufficient linearity (R^2^ > 0.999 with a regression equation of y = 45.993x + 6.503 in the concentration range of 0.5 to 25 µg/mL for BCT and R^2^ > 0.999 with a regression equation of y = 130.480x − 51.984 in the concentration range of 1 to 25 µg/mL for TRT), high sensitivity (LOQ of 0.5 µg/mL and 1 µg/mL for BCT and TRT, respectively and LOD of 0.17 µg/mL and 0.33 µg/mL for BCT and TRT, respectively), adequate precision (with CV% of < 15%), and acceptable accuracy values (85–115%).

### BCT-TRT loaded niosome optimization

Before the optimization process, various percentages of each drug, including 5%, 2.5%, 2%, and 1.5% of the mass of total lipid, were utilized for niosomes preparation. Due to drug expulsion occurrence and an increase in particle size of the niosomes with higher drug content, finally, the niosomes containing 1.5%w/w of BCT and TRT were selected. This percentage was kept fixed in all suggested runs by Design-Expert.

**Figure 1:**
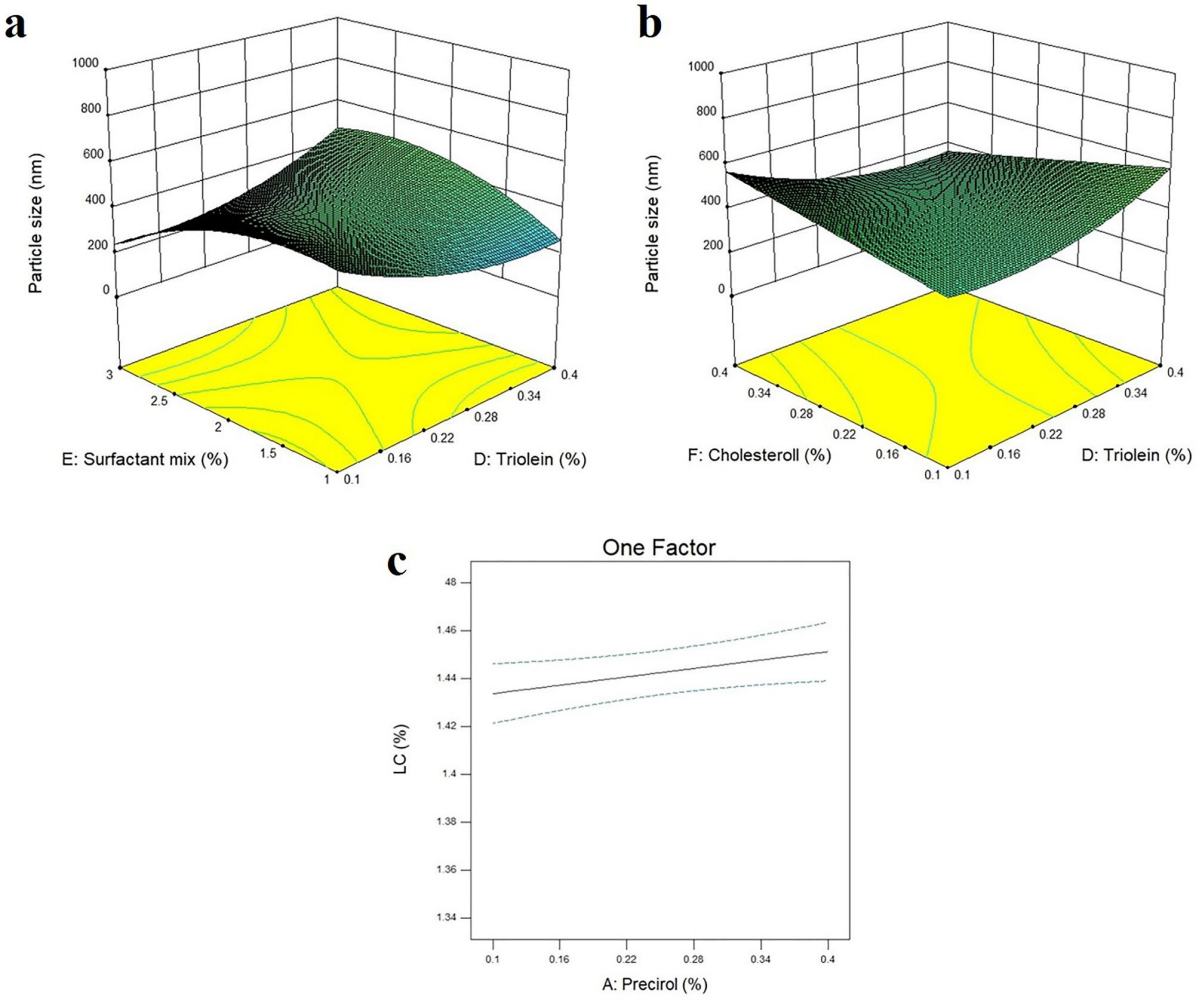
(**a**) The 3D-graph of the binary effect of the percentages of triolein and surfactants on the particle size of the niosomes, (**b**) The 3D-graph of the binary effect of the percentages of triolein and cholesterol on the particle size of the niosomes, and (**c**) The one-factor plot of the effect of the percentage of Precirol on drug loading capacity of the niosomes.

Results of niosomes optimization based on Design-Expert software are summarized in Table 1. As it is obvious, the prepared niosomes in these 38 runs had a particle size range of 64–964 nm and a loading capacity (%LC) range of 1.36 to 1.47%. The suggested model by Design-Expert was the Quadratic model with an R-squared value of 0.848. This model was completely fit with a non-significant lack of fit of 0.318. However, the P-value of the suggested model was not significant (*P* value = 0.115) for untreated data but it became significant through the logarithmic transformation (*P* value = 0.033). The results can be found in Supplementary Table S1 online. As shown in Fig. 1a,b, among all assessed independent variables, the concurrent effects of triolein and surfactants (Brij 35 and Span 60) and also triolein and cholesterol were significant on the particle size of the prepared niosomes with *P* values of 0.012 and 0.029, respectively. In addition, only the percentage of Precirol in the lipid matrix of niosomes showed a significant effect (*P* value of 0.036) on drug loading capacity (Fig. 1c).

**Table 1:** Different suggested runs by Design-Expert and the obtained results regarding niosomes’ particle size and loading capacity.

Run	Independent variables						Dependent variables	
	%Precirol (range: 0.1–0.4%w/v)	%Stearic acid (range: 0.1–0.4%w/v)	%Capryol PGMC (range: 0.1–0.4%w/v)	%Triolein (range: 0.1–0.4%w/v)	%Surfactants mixture (range: 1–3%w/v)	%Cholesterol (range: 0.1–0.4%w/v)	Particle size (nm)	%Loading capacity
1	0.30	0.21	0.10	0.10	2.00	0.39	653.00	1.46
2	0.10	0.40	0.10	0.40	3.00	0.10	964.00	1.36
3	0.10	0.40	0.10	0.14	1.00	0.40	409.00	1.44
4	0.40	0.40	0.39	0.40	3.00	0.10	626.00	1.42
5	0.10	0.36	0.21	0.10	3.00	0.40	476.00	1.45
6	0.40	0.40	0.40	0.10	2.00	0.40	540.00	1.44
7	0.26	0.40	0.21	0.13	2.00	0.12	564.00	1.43
8	0.10	0.10	0.40	0.40	3.00	0.40	540.00	1.43
9	0.40	0.10	0.10	0.40	1.00	0.40	341.00	1.46
10	0.12	0.40	0.38	0.28	2.00	0.25	549.00	1.41
11	0.10	0.40	0.10	0.33	3.00	0.35	331.00	1.45
12	0.27	0.23	0.40	0.14	3.00	0.23	351.00	1.45
13	0.39	0.29	0.38	0.23	2.00	0.10	523.00	1.46
14	0.10	0.18	0.28	0.25	3.00	0.10	319.00	1.44
15	0.40	0.40	0.10	0.25	3.00	0.29	359.00	1.46
16	0.12	0.40	0.38	0.28	2.00	0.25	896.00	1.44
17	0.17	0.12	0.10	0.17	1.00	0.13	571.00	1.43
18	0.40	0.23	0.25	0.14	1.00	0.22	375.00	1.41
19	0.10	0.20	0.26	0.40	1.00	0.17	292.00	1.40
20	0.22	0.22	0.36	0.25	1.00	0.40	322.00	1.45
21	0.25	0.38	0.22	0.40	2.00	0.40	419.00	1.45
22	0.39	0.40	0.40	0.40	1.00	0.16	389.00	1.45
23	0.40	0.19	0.40	0.40	2.00	0.30	463.00	1.46
24	0.33	0.10	0.40	0.29	1.00	0.10	369.00	1.45
25	0.40	0.10	0.27	0.23	3.00	0.40	324.00	1.45
26	0.10	0.17	0.10	0.30	2.00	0.37	370.00	1.44
27	0.40	0.10	0.10	0.10	3.00	0.10	108.00	1.45
28	0.22	0.22	0.36	0.25	1.00	0.40	274.00	1.46
29	0.26	0.40	0.21	0.13	2.00	0.12	448.00	1.45
30	0.10	0.30	0.10	0.10	3.00	0.10	64.00	1.44
31	0.39	0.26	0.11	0.35	2.00	0.10	376.00	1.46
32	0.27	0.10	0.18	0.40	3.00	0.23	368.00	1.43
33	0.10	0.29	0.40	0.10	1.00	0.10	539.00	1.44
34	0.24	0.40	0.19	0.36	1.00	0.10	519.00	1.47
35	0.10	0.10	0.33	0.10	2.00	0.30	417.00	1.47
36	0.28	0.28	0.10	0.28	1.00	0.31	313.00	1.45
37	0.27	0.10	0.18	0.40	3.00	0.23	407.00	1.46
38	0.40	0.23	0.25	0.14	1.00	0.22	387.00	1.46

Final equations for particle size and loading capacity, in terms of coded factors, are presented in Eqs. (1) and (2). According to Eq. (1) a positive correlation was obvious between particle size and concentration of different ingredients including stearic acid, Capryol PGMC, triolein, and cholesterol. While a negative correlation was observed between the concentration of Precirol and surfactant mixture and the particle size of the niosomes. Based on Eq. (2), a positive correlation was seen between %LC and concentration of Precirol, Capryol PGMC, and cholesterol, while a negative correlation was observed between the concentration of stearic acid, triolein, and surfactant mixture and %LC of the niosomes.

1 $$ \begin{aligned} {\varvec{Log}}_{{{\mathbf{10}}}} \left( {{\varvec{Particle}}\, \, {\varvec{size}}} \right) \, = & + \,2.600 \, - \, 0.021*A \, + \, 0.070*B \, + \, 0.040*C \, + \, 0.017*D \, - 6.037E \\ & - \,003*E \, + \, 6.696E - 003*F \, - \, 0.028*AB \, - \, 0.035*AC \, - \, 9.763E \\ - \,003*AD \, - \, 0.019*AE + \, 0.020*AF \, + \, 0.049*BC \, + \, 4.952E - 005*BD \\ + \, \, 0.052*BE \, - \, 0.053*BF \, - \, 7.689E - 003*CD + \, 0.069*CE \, \\ - \, \, 0.012*CF \, + \, 0.134*DE \, - \, 0.099*DF \, + \, 0.083*EF \\ \end{aligned} $$

2 $$ \begin{aligned} {\varvec{Log}}_{{{\mathbf{10}}}} \left( {{\varvec{Loading}} \, \,{\varvec{capacity}}} \right) = & + 0.159 \, + 2.672E - 003*A \, - \, 1.241E - 003*B \, + \, 5.052E - 004*C \\ & - \, 1.813E - 003*D \, - \, 6.005E - 004*E \, + \, 2.204E - 003*F \\ \end{aligned} $$

while A, B, C, D, E, and F stand for Precirol, Stearic acid, Capryol PGMC, Triolein, surfactant mixture, and Cholesterol, respectively.

### Formulation characterization

According to the results of optimization, formulation 14 (F14) with the desired drug loading capacity and particle size of about 300 nm, which was promising for follicular targeting purposes^24^, was selected as the optimum formulation, and further characterizations including particle size, zeta potential, stability, drug loading, TEM, and DSC were performed on this optimum sample. The exact amounts of drug, surfactants, and lipid matrices of F14 as the optimum niosomal formulation are summarized in Table 2. Furthermore, particle size targeting (target size of 300 nm) through the Design-Expert software, revealed a desirability of 0.948 as shown in Supplementary Fig. S1 online.

**Table 2:** The exact amounts of the contents of the optimum niosomal formulation (F14).

BCT^a^	TRT^b^	BHT^c^	Stearic acid	Cholesterol (mg)	Precirol	Triolein	Capryol PGMC	Brij 35	Span 60	Organic phase	PBS^d^
1.36 mg	1.36 mg	4.55 mg	18.25 mg	10.00 mg	10.00 mg	25.39 mg	28.15 mg	150 mg	150 mg	10 ml	20 ml

^a^Bicalutamide, ^b^Tretinoin, ^c^Butylated hydroxytoluene, ^d^Phosphate buffered saline.

#### Particle size, size distribution, and zeta potential

**Figure 2:**
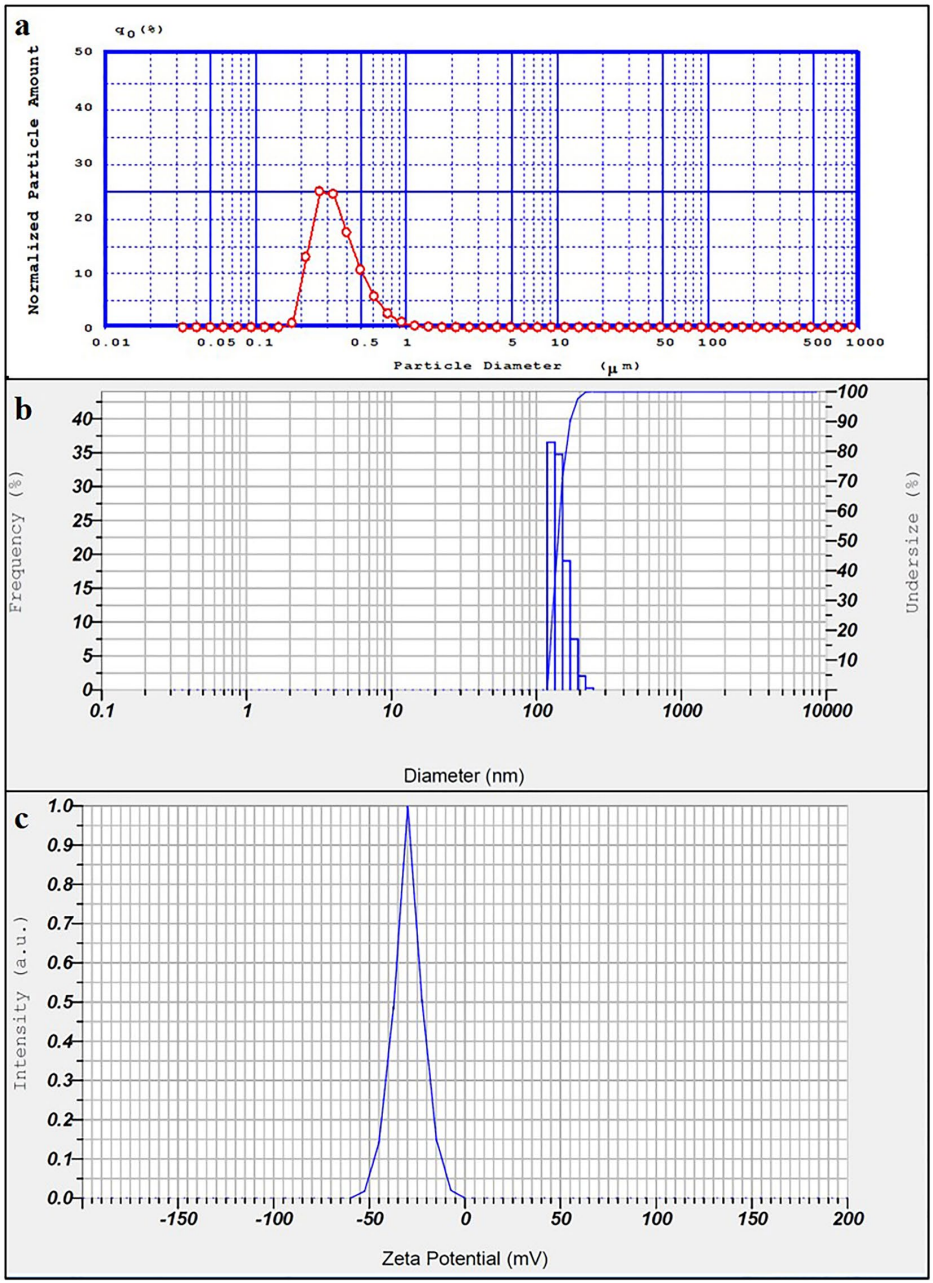
(**a**) PSA graph of BCT–TRT loaded niosomes with an average diameter of 319.20 ± 18.50 nm and span index of 1.07, (**b**) DLS graph of BCT–TRT loaded niosomes with an average diameter of 238.30 ± 8.80 nm and polydispersity index (PDA) of 0.61, and (**c**) Zeta potential of BCT-TRT loaded niosomes (− 29.70 ± 0.36 mV).

Results of particle size and size distribution assessment, as depicted in Fig. 2a,b, revealed that the prepared niosomes were homogenous in particle size with an average diameter of 319.20 ± 18.50 nm (using PSA technique) and 238.30 ± 8.80 nm (using DLS technique). The calculated span index and polydispersity index (PDI) in these two techniques were 1.07 and 0.61, respectively.

Results of zeta potential assessment revealed that the optimum formulation had a zeta potential of − 29.70 ± 0.36 mV (Fig. 2c). This negative zeta potential and further electrostatic repulsion among the nanoparticles could enhance desired physical stability for the prepared niosomal formulation through the prevention of nanoparticle aggregation.

#### Physical stability

**Figure 3:**
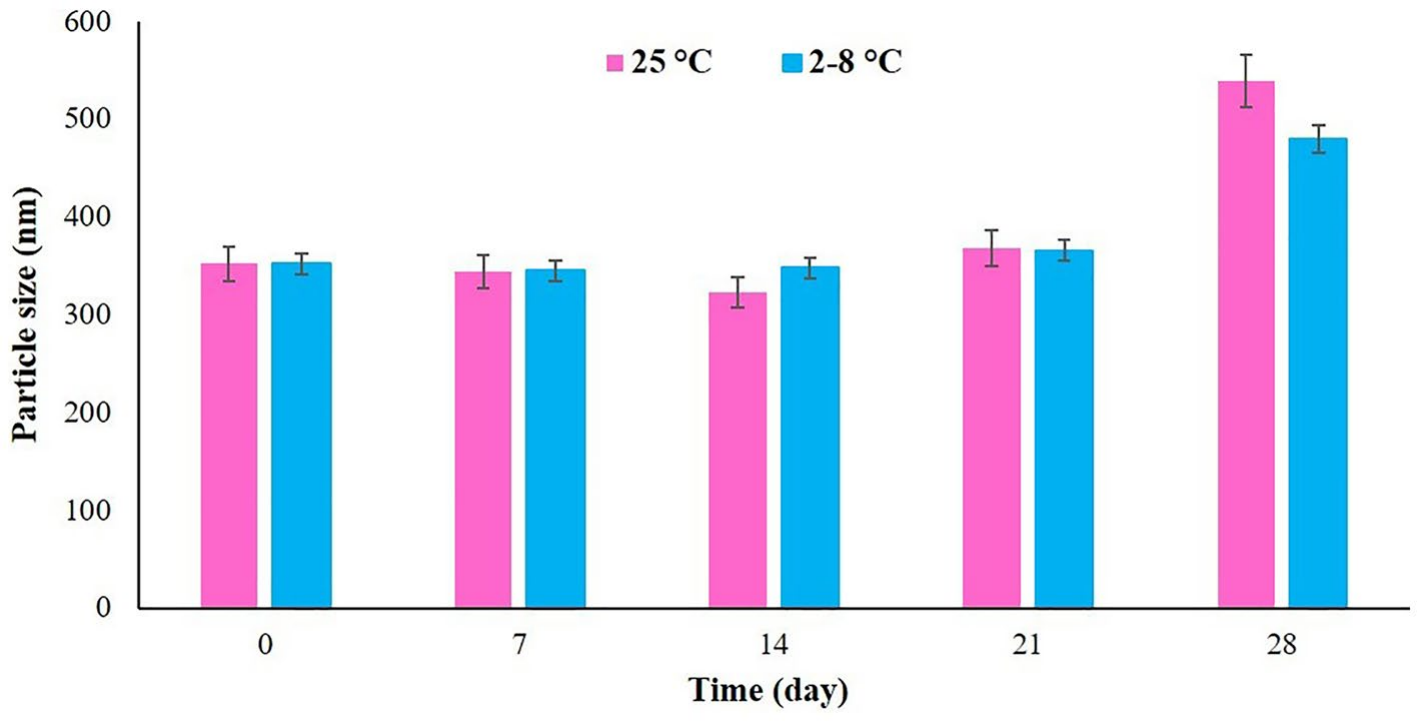
Stability assessment of the optimum BCT-TRT loaded niosome (F14) at room temperature (25 °C) and refrigerator (2–8 °C) at one-week intervals up to 28 days after formulation preparation.

As shown in Fig. 3, the physical stability assessment of the optimum formulation (F14) at room temperature (25 °C) and refrigerator (2–8 °C) revealed that the niosomes were stable for up to 21 days without significant particle size enhancement and drug expulsion. However, after 28 days of storage, despite of lack of drug expulsion, an enhancement in the particle size of the niosomes was seen.

#### Drug loading

The results of the indirect drug loading assessment are summarized in Table 3. The high %EE values of 94.63% and > 99% for BCT and TRT, respectively, which can be attributed to the lipophilic nature of these drugs. In addition, based on the results LC of prepared niosomes was 1.40% and 1.48% for BCT and TRT, respectively in the optimum formulation. Although the obtained values for %LC for both BCT and TRT are relatively low, however, due to the high potency of these drugs, the obtained percentages would be clinically sufficient for acne management (TRT concentration in conventional products is about 0.025–0.1%).

**Table 3:** Entrapment efficiency and loading capacity of BCT-TRT loaded niosomes in optimum formulation (F14).

	%Loading capacity ± SD	%Entrapment efficiency ± SD
TRT^a^	1.48 ± 0.00	> 99%
BCT^b^	1.40 ± 0.01	94.63% ± 0.50

^a^Tretinoin, ^b^Bicalutamide.

#### Transmission electron microscopy

**Figure 4:**
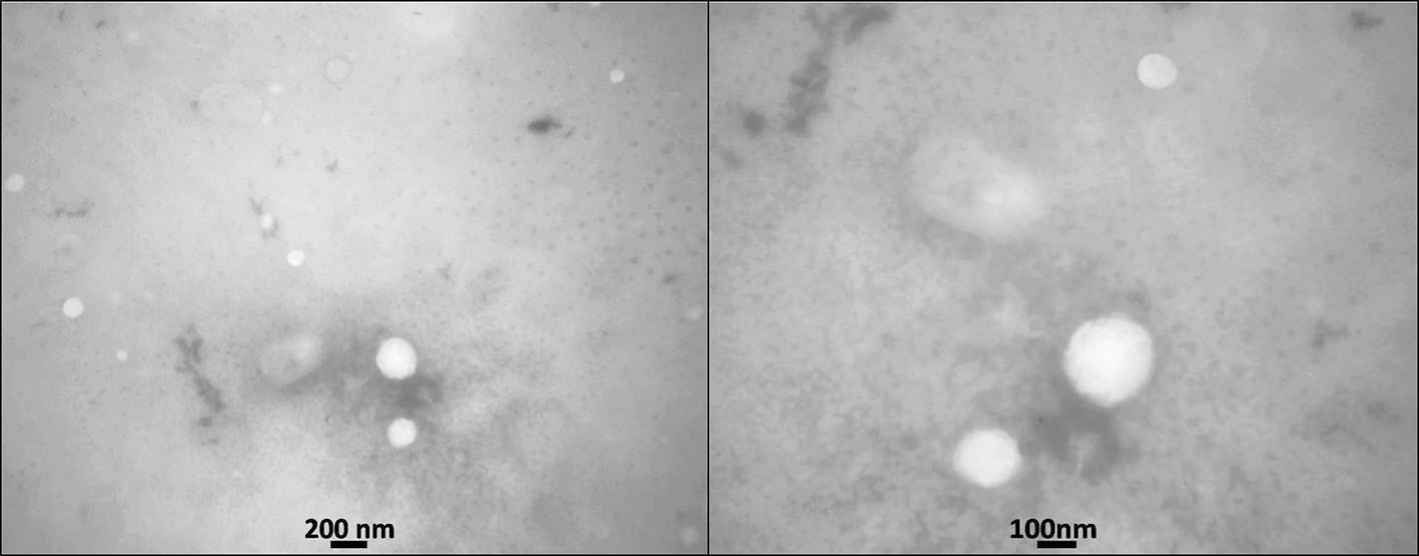
Transmission electron microscopy of BCT-TRT loaded niosomes (F14).

Transmission electron microscopy assessment results (Fig. 4) revealed that the optimum BCT-TRT loaded niosomes were spherical in shape and homogenous in size. In addition, TEM analysis results were compatible with the data obtained through the PSA or DLS techniques.

#### Differential scanning calorimetry

**Figure 5:**
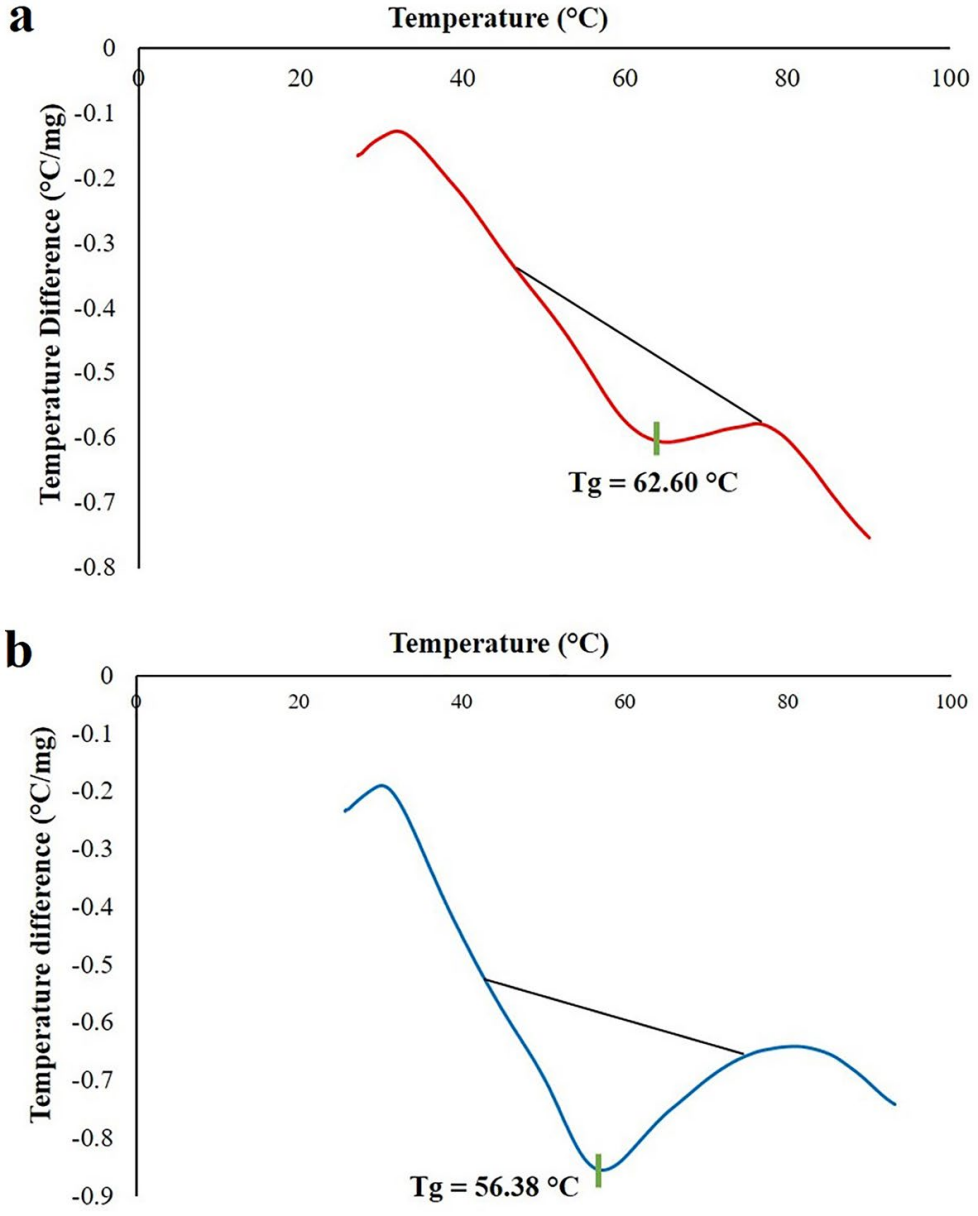
Differential scanning calorimetry (DSC) of (**a**) drug-free niosomes and (**b**) BCT-TRT loaded niosomes.

Results of DSC thermal analysis (Fig. 5) revealed that encapsulation of both BCT and TRT within the niosomes structure was accompanied by a reduction in glass transition temperature (T_g_) from 62.60 to 56.38 °C. Also, the DSC thermograms of BCT, TRT, and all ingredients used in niosome preparation can be found in Supplementary Figs. S2, S3 online.

### In vivo* follicular targeting*

**Figure 6:**
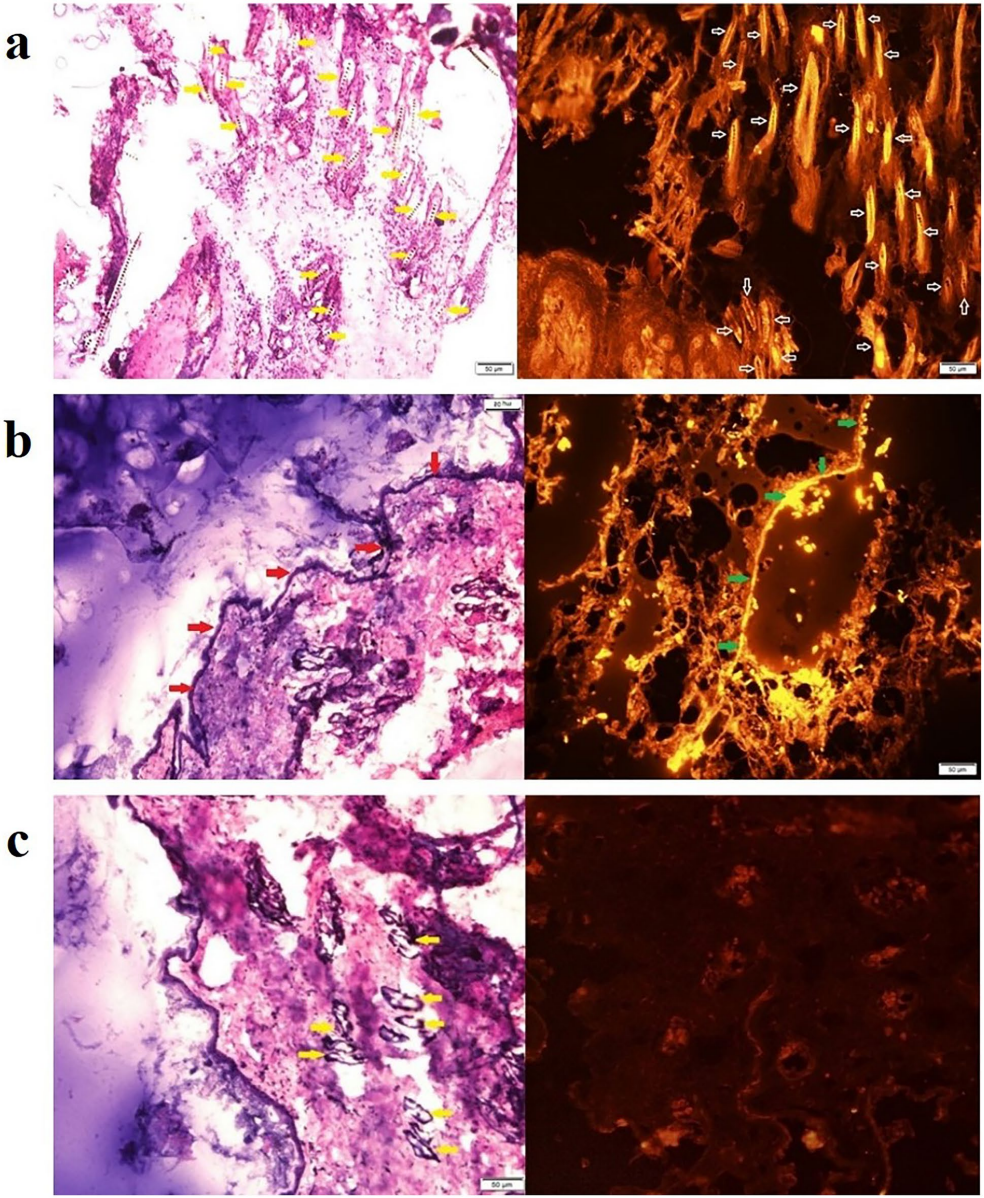
(**a**) Rhodamine B-loaded niosomes gel with an average diameter of 300 nm (× 100), a high-intensity fluorescent dye was obvious within the hair follicles, hair follicles are marked with yellow and white arrows; (**b**) Conventional rhodamine B gels (× 100), a sharp fluorescent lumination was obvious on the surface of the epidermis, the epidermis surface have been shown with red and green arrows; (**c**) Negative control (skin without formulation application) (× 100), the hair follicles are shown with yellow arrows. The left figures were obtained from the optical microscopy of H and E staining slides and the right figures were obtained from the fluorescent microscopy of the frozen slides.

As shown in Fig. 6, the results of in vivo follicular targeting and fluorescent microscopy assessments revealed that although the application of rhodamine B as conventional gel formulation failed to penetrate through skin layers and mainly accumulated at the epidermal surface and induced fluorescent illumination on the surface of the stratum corneum layer, however, rhodamine B-loaded niosomes gel significantly target the hair follicles and induce high-intensity fluorescent dye within the pilosebaceous units. In addition, the results obtained from these formulations were compared with negative control (the skin without formulation application) to rule out the possibility of skin or hair auto-fluorescence characteristics as shown in Fig. 6. Therefore, it seems that niosomes with an average diameter of about 300 nm are promising nanocarriers for follicular targeting in order to manage androgenetic skin disorders including hirsutism, acne, and alopecia.

## Discussion and conclusion

To the best of our knowledge to date, there is no published data in simultaneous detection and quantification of BCT and TRT in pharmaceutical matrices. Since BCT and TRT have different λ_max_ of 272 nm and 350 nm, respectively, simultaneous detection and quantification of these two drugs are impossible through conventional UV detectors, therefore we recruited the PDA detector for simultaneous analysis of BCT and TRT in pharmaceutical matrices. The validated RP-HPLC method with PDA detector passed the FDA guideline criteria for method validation^25^.

During the optimization process, the niosome with desired particle size and drug loading capacity was selected as the optimum formulation (F14). The optimum particle size of 300 nm was obtained from the previous studies on trans-follicular drug delivery^12^. In this regard, the results of our previous study on cyproterone acetate-loaded NLCs revealed that nanoparticles with an average diameter of 300 nm significantly target the hair follicle in comparison to the larger and smaller particles^24^. Due to the different nature of the NLCs in comparison to niosomes then it seems that follicular targeting is mainly size-dependent and in this regard, the result of this study would be applicable to the nano-based delivery systems having the same size ranges especially nanoparticles with lipid-based natures. Moreover, results of a recent study on finasteride-loaded niosomes with an average diameter of 260 nm revealed a 20-fold higher drug accumulation within the hair follicles in comparison to the hydroethanolic solution of finasteride^14^.

Although the suggested model by Design-Expert for particle size optimization was not significant for untreated data, however, after the application of logarithmic transformation, the suggested model (2FI model) became significant and also the lack of fit was not significant which confirmed the fitness of the model for particle size prediction. However, the suggested model for %LC was still not significant after logarithmic transformation. This non-significant model for %LC can be attributed to the primary pilot screening test that was performed before the inclusion of data to Design-Expert. In this regard, various percentages of BCT and TRT were tested to finally reach to the optimum percentage of 1.5% of the mass of the lipid matrix for each drug. After that, all suggested runs by Design-Experiment were performed with this optimum percentage of BCT and TRT and therefore resulted in a high %EE of > 95% and %LC of 1.36 to 1.47% in all these 38 runs. Consequently, the suggested model was not significant and only the Precirol concentration could significantly affect the %LC of the prepared niosomes. Results of a previous study on the optimization of topical methotrexate-loaded niosomes through the Design-Expert software suggested a significant 2FI model for particle size which was compatible with the results of the current study^26^.

According to the optimization results, the stearic acid concentration, the concurrent effect of triolein and surfactant mixture concentrations, cholesterol and surfactant mixture concentrations, and also triolein and cholesterol concentrations could significantly affect the niosomes’ particle size. These findings of the current study were compatible with the results of a previous study on nevirapine-loaded niosomes optimization. Niosomal nevirapine formulation was optimized through the design of experiment and the response surface methodology and the results confirmed the significant effect of cholesterol and surfactant contents on particle size and %EE of the prepared niosomes^27^. Therefore, the combined effect of lipid matrix and surfactants can profoundly affect the niosomes particle size^27^. In addition, results of another study on niosomal testosterone enanthate (testosome) revealed that higher concentrations of cholesterol lead to the fabrication of vesicles with larger particle sizes^28^ which was in agreement with the findings of the current study. The significant effect of triolein on niosomes’ particle size was comparable with the results of our previous studies on NLCs. In this regard, an increase in triolein concentration up to an optimum level could lead to a decrease in nanoparticles’ size and after that optimum level, the relation became reversed. In addition, triolein could reduce nanoparticles’ polydispersity through the reduction in the span indices^29^. Results of the current study revealed that among the assessed independent variables, there was a significant positive correlation between Precirol concentration and %LC of both BCT and TRT within the niosomes. These findings were comparable with the results of a previous study on BCT-loaded NLCs in which the presence of Precirol, as a highly lipophilic agent with HLB of 2, could significantly enhance the encapsulation of BCT (as a lipophilic drug) within the NLCs structure^30^.

The optimum formulation of BCT-TRT loaded niosomes (F14) with the desired particle size of about 300 nm were homogenous in size and had spherical shapes (Fig. 4). The spherical shape of prepared niosomes was confirmed through TEM analysis and it was compatible with the results of the recent studies on human growth hormone-loaded niosomes^31^, simvastatin-loaded niosomes, and also Epigallocatechin Gallate niosomal nanocarriers that were spherical in shape^32^. The negative zeta potential of − 29.70 ± 0.36 mV could result in adequate physical stability of the prepared niosomes through the induction of electrostatic repulsion and avoidance of nanoparticle aggregation during the storage. This negative zeta potential was the same as the previous studies on pilocarpine hydrochloride-loaded niosomes containing Span 60 as a non-ionic surfactant^33^. In addition, the results of another study on topical delivery of cyclosporine-loaded niosomes showed a negative zeta potential for the prepared niosomes^34^. Moreover, ascorbic acid-loaded hyaluronic acid-coated niosomes with negative zeta potential (− 38.70 mV) showed the greatest potential in skin penetration enhancement.

The stability results revealed that the optimum formulation was physically stable for up to 3 weeks (both at room temperature (25 °C) and refrigerator (2–8 °C)). Also, no drug expulsion occurred during the four-week storage stability assessment. These results were compatible with the results of a previous study on melatonin-loaded niosomes which showed a small change in particle size during four weeks of stability assessment^35^. Moreover, adapalene-loaded niosomes, with an average diameter of 278 nm that were designed and characterized for acne treatment, were stable during the one month of physicochemical stability assessment^36^.

Due to the lipophilic characteristics of both BCT and TRT with log P of 2.3 and 6.3, respectively, high entrapment efficiency values of 94.63% and > 99%, were predictable. These results were completely compatible with the results of the previous studies on tretinoin-loaded niosomes with %EE values of 90.51–98.30% and also tretinoin-loaded diolein-niosomes with %EE of almost 100%^20,37^. Moreover, according to the previous studies, the high %EE of TRT within the unilamellar niosmes could significantly enhance the photostability of the loaded TRT in comparison to the methanolic solution of TRT^18^. Furthermore, results of another study showed %EE of 73.58% for BCT-loaded NLCs^38^, therefore, it seems that niosomal formulation could enhance %EE of BCT in comparison to the NLC formulation.

Results of DSC thermograms showed that BCT and TRT encapsulation within the niosomes could influence the T_g_ value of the optimum niosomal formulation (T_g_ value was reduced from 62.60 to 56.38 °C after drug loading).

According to the results of the current study, rhodamine B-loaded niosomes with an average diameter of about 300 nm could efficiently target the hair follicles with a sharp fluorescent light illumination within the pilosebaceous units. The obtained result was comparable to our previous study on cyproterone acetate-loaded NLCs in which nanoparticles with an average diameter of 300 nm showed better follicular targeting potential with more fluorescent illumination in comparison to the smaller or larger nanoparticles and microparticles^24^. Results of another study on doxycycline-loaded niosomes revealed that the optimum formulation with an average particle size of 362.88 nm could successfully target hair follicles^39^ which was in agreement with the results of the current study. Furthermore, it has been reported that superoxide dismutase-loaded niosomes with a particle size range of 152–325 nm showed enhanced follicular targeting^40^. Also, it has been reported that according to the cuticle thickness of the human hairs, nanoparticles with a particle size range of 300–600 nm can penetrate more efficiently through the trans-follicular pathway and therefore, deeper deposition within the hair follicles can be achieved^41^. Moreover, the results of a recent study on finasteride-loaded niosomes with a mean particle size of 260 nm showed efficient follicular targeting with a 20-fold increment in drug accumulation within the hair follicles in comparison to the hydroethanolic solution of finasteride^14^.

In conclusion, a novel dual drug delivery system consisting of BCT-TRT loaded niosomes with an average diameter of about 300 nm was designed, optimized, and characterized. Both BCT and TRT were analyzed simultaneously through a validated HPLC–PDA technique. The optimum niosomal formulation showed desired particle size and negative zeta potential. The prepared niosomes were homogenous in size and spherical in shape with high encapsulation efficiency for both BCT and TRT. The in vivo animal study results revealed that rhodamine B-loaded niosomes with particle size of 300 nm could profoundly target hair follicles and pilosebaceous units. Therefore, the designed niosomal BCT and TRT co-delivery system would be promising in acne vulgaris management with follicular targeting potential.

The main limitations of the niosomal delivery systems are their low physical stability profile compared to conventional formulations, low loading capacity, and need to more sophisticated technique of production.

## Methods

All experiments were performed in accordance with relevant guidelines and regulations.

### Materials

Cholesterol, Stearic acid, rhodamine B, butylated hydroxytoluene (BHT), Tween 80, and *O*-phosphoric acid were purchased from Merck (Darmstadt, Germany). Triolein, Span 60, and Brij 35 were obtained from Sigma (St. Louis, Missouri, USA). Capryol PGMC and Precirol were purchased from Gattesosse (France). Carbomer 934 was purchased from BF Good Reach (Charlotte, USA). Standard powder of Tretinoin (TRT) with 99.9% purity was kindly gifted by Pars Darou Pharmaceutical Co., and Bicalutamide (BCT) was extracted from tablets (Iran Hormon Pharmaceutical Co., Iran), and its purity was checked by the validated HPLC technique. Acetone, ethanol, chloroform, methanol, and acetonitrile were HPLC grade and were purchased from Merck.

### Statistical analysis

In this study, statistical analysis was performed using Design-Expert software (version 10.0.7, Stat-Ease Inc., Minneapolis, USA) and SPSS software (version 26), and *P* value < 0.05 was considered as significant.

### Quantitative assessment of BCT and TRT

BCT and TRT were analyzed simultaneously through a validated reverse-phase high-performance liquid chromatography (RP-HPLC) technique with A photodiode array (PDA) detector (HPLC Knauer, Azura, DAD 2.1L, Germany). In this regard, the stationary phase was an HPLC column (Eurospher 100-5 C18, Knauer, Germany, 250 × 4.6 mm) and the mobile was composed of acetonitrile: phosphate buffer (75:25%v/v ratio). The λ_max_ for BCT and TRT were set at 272 nm and 350 nm, respectively. The sample volume was 20 µL, the flow rate was set 1 ml/min, and the column temperature was fixed at 25 °C. The stock solutions for both BCT and TRT had a concentration of 1 mg/mL, while the working solutions had a concentration of 100 µg/mL. Standard solutions were prepared in concentration ranges of 1–25 µg/mL and 0.5–25 µg/mL for BCT and TRT, respectively.

### BCT-TRT loaded niosome preparation

BCT-TRT loaded niosomes were prepared through a modified thin film hydration technique^37,42^. In this regard BCT, TRT, BHT, surfactants including Brij 35 and Span 60, and lipid matrices containing stearic acid, cholesterol, Precirol, triolein, and Capryol PGMC were dissolved in 10 ml of ethanol: chloroform mixture (1:1%v/v ratio) in a round-bottom flask. The solvent was evaporated and a thin film was formed through a rotary evaporator (IKA HB10, Germany) at 55 °C in a water bath and a stirring rate of 150 rpm. The thin film was dried completely under the low pressure of a vacuum pump. After that, the prepared thin film was hydrated through the addition of 20 ml phosphate buffered saline (PBS, pH = 7.4). The resuspended sample was heated up to 55 °C in a water bath at a stirring rate of 150 rpm for 30 min to form niosomal suspension. In the end, the prepared niosomes underwent a three-cycle round of probe sonication (15 min sonication for each cycle with 5 min off between cycles). Due to the light-sensitivity of TRT and possible decomposition, all of these processes were accomplished within aluminum foil-wrapped glassware.

### BCT-TRT loaded niosome optimization

BCT-TRT loaded niosome optimization was performed through the Design-Expert software (version 10.0.7, Stat-Ease Inc., Minneapolis, USA), according to response surface optimal design, based on our previous report^29^. In this regard, the percentages of surfactants (with a level of 1–3%w/v containing Brig 35 and Span 60 with 1:1%w/w ratio and HLB_M_ = 10.8) and lipid matrices including stearic acid, cholesterol, Precirol, triolein, and Capryol PGMC (with a level of 0.1–0.4% w/v for each one) were selected as independent variables and particle size and loading capacity were considered as dependent variables. Based on these variables, the software suggested 38 runs as shown in Table 1. Before the optimization process, the optimum percentage of BCT and TRT that can be loaded within niosomal formulation was assessed through the assessment of drug expulsion and potential increment in particle size during storage. In this regard, during the early pilot study, various percentages of each drug including 5%, 2.5%, 2%, and 1.5% w/w of the mass of lipid mixture were assessed to obtain the desired BCT-TRT loaded niosome without drug expulsion. Finally, the obtained optimum percentage of BCT and TRT was used for all suggested runs by Design-Expert.

### Formulation characterization

According to the formulation optimization results, the optimum sample with desired particle size and loading capacity was selected and further formulation characteristics including particle size, zeta potential, drug loading physical stability, particle morphology, and differential scanning calorimetry were assessed for that optimized niosomal formulation.

#### Particle size, size distribution, and zeta potential assessment

Particle size and size distribution were assessed using the particle size analyzer (PSA, Shimadzu, SALD-2101, Japan) and dynamic light scattering (DLS, SZ-100, Horiba, Germany). Furthermore, to assess the polydispersity of the prepared niosomes span index was calculated according to Eq. (3) from the PSA results, also the polydispersity index was reported by DLS. In addition, the zeta potential of niosomes was measured through DLS (SZ-100, Horiba, Japan) to confirm niosomes physical stability.

3 $$ Span\, index = \frac{{D_{90} - D_{10} }}{{D_{50} }} $$

where D_90_, D_50_, and D_10_ are 90%, 50%, and 10% under-sized particle sizes, respectively.

#### Physical stability assessment

The physical stability of the prepared niosomes was assessed through the extensive particle size change and also visual drug expulsion assessment up to 28 days, at least for one-week intervals, both at room temperature (25 °C) and refrigerator (2–8 °C).

#### Drug loading assessment

In order to assess the drug loading, entrapment efficiency (%EE) and loading capacity (%LC) were considered, using centrifugation ultrafiltration technique. In this regard, 5 ml of the prepared niosomes was poured into the upper chamber of the Amicon filter tube (MWCO 3KDa, Amicon Ultra-4, Millipore Co., MA, USA) and the sample was centrifuged at 4000 rpm for 15 min. Then, the filtrate was assessed through a validated HPLC–PDA method to quantify the unloaded drug portion in the samples and the %EE and %LC were calculated through Eqs. (4), (5), respectively.

4 $$ \% EE = \frac{Laoded\, drug}{{Total \,drug}} \times 100 $$

5 $$ \% LC = \frac{Loaded \,drug}{{Loaded\, drug + total\, lipid + total \,surfactant}} $$

#### Transmission electron microscopy (TEM) assessment

Particles morphology and particle sizes of BCT–TRT loaded niosomes were assessed through TEM (EM10C, Zeiss, Germany). In this regard, 20 µL of the sample was placed on a 300 Mesh Formvar carbon-coated copper grid (EMS, USA) for 2 min. Then, in order to visualize the niosomes, the negative staining process was performed using a 2% aqueous solution of uranyl acetate for 1 min. After that, the copper grid was air dried and TEM was performed at an accelerating voltage of 100 kV.

#### Differential scanning calorimetry (DSC) assessment

Differential scanning calorimetry (DSC-Q600, IndiaMart, India) was performed both for drug free-niosomes and BCT-TRT loaded niosomes. In this regard, fifty microliters of each sample was utilized. The scan rate was 10 °C/min and samples were heated from 25 up to 90 °C. DSC process was performed on air atmosphere.

### Rhodamine B-loaded niosome gel preparation

Rhodamine B, as a fluorescent dye, was used to assess the possible prepared niosomes follicular targeting^24^. In this regard, rhodamine B-loaded niosomes with the same concentration as each BCT and TRT and also desired particle size were prepared through the aforementioned thin film hydration technique. After that, the niosomal gel formulation was fabricated through the addition of sodium carboxymethyl cellulose (Na CMC, 2%w/v) and Carbomer 934 (0.5%w/v) to the freshly prepared niosomes. Finally, the pH-dependent gel formation occurred through the addition of sodium hydroxide (NaOH, 1 N) to the hydrated polymers.

### Animal study and ethics

Male golden hamsters were obtained from the Center of Experimental and Comparative Medicine at Shiraz University of Medical Sciences, Shiraz, Iran. The golden hamsters, with a weight range of 100–150 g, were kept in a relative humidity of 40% and temperature of 25 °C. Animals were handled according to the protocol of the Ethics Committee of Shiraz University of Medical Sciences, Shiraz, Iran, with ethics code No. IR.SUMS.AEC.1401.065, and approval date of 08.17.2022. The reporting in the manuscript follows the recommendations in the ARRIVE guidelines.

#### In vivo* follicular targeting assessment*

To assess follicular targeting, 6 male hamsters were divided into 3 groups (2 hamsters in each group). At first, hamsters were anesthetized using ketamine (70 mg/kg) and xylazine (10 mg/kg) cocktail. Then, the hamsters’ flank organ skin was shaved using an electric shaver (Moser, Germany). Rhodamine B-loaded niosome gel was applied on the flank organ skin of the first group, rhodamine B conventional gel with equal concentration was applied for the second group, and the third group was considered as the negative control without formulation application to assess the possible skin or hairs’ auto-fluorescence characteristics. After the formulation application to the aforementioned site of the animal skin, a gentle massage was applied to facilitate the follicular targeting. After 2 h of formulation application, a skin biopsy was obtained from each hamster. In the end, the animals were sacrificed through the thiopental (70 mg/kg) administration. The obtained skin biopsies were kept in an aluminum foil and transferred to the pathology lab. Then, the frozen sections (with a thickness of 3 µm) were prepared from the collected skin samples using Cryocut instrument (Leica, Cryocut 1800, USA). In addition, the prepared slides were stained with hematoxylin and eosin (H and E) to visualize the borders of the cells. Finally, frozen slides were evaluated using the fluorescence microscopy technique (Fluorescent microscope, Olympus, BX51, Germany, with λ_ex_ and λ_em_ of 553 and 627 nm, respectively). Moreover, the H and E slides were evaluated through the optical microscopy method. This in vivo assessment was used to signify the predominant route of penetration and deposition within skin and/or skin organelles through the recruitment of rhodamine B as a fluorescent marker.

### Supplementary Information

Supplementary Information.

## Data Availability

The data that support the findings of this study are available from [Soliman Mohammadi-Samani] upon request.
