# Supplementary material for: Optimization, characterization, and follicular targeting assessment of tretinoin and bicalutamide loaded niosomes
Source: Sci Rep. 2023 Nov 16;13:20023. doi: 10.1038/s41598-023-47302-6 (PMC10654571; doi:10.1038/s41598-023-47302-6)
Supplement: Supplementary file 1 — Supplementary Information. [file 41598_2023_47302_MOESM1_ESM.pdf]

Optimization, Characterization, and Follicular Targeting Assessment of Tretinoin and Bicalutamide-Loaded Niosomes

Parisa Ghasemiyeh, Fatemeh Moradishooli, Saeid Daneshamouz, Reza Heidari, Uranous Niroumand and Soliman Mohammadi-Samani*

*Corresponding author: Soliman Mohammadi-Samani (smsamani@sums.ac.ir), Pharm.D., Ph.D., Professor of Pharmaceutics, School of Pharmacy, Shiraz University of Medical Sciences, Shiraz, Iran.

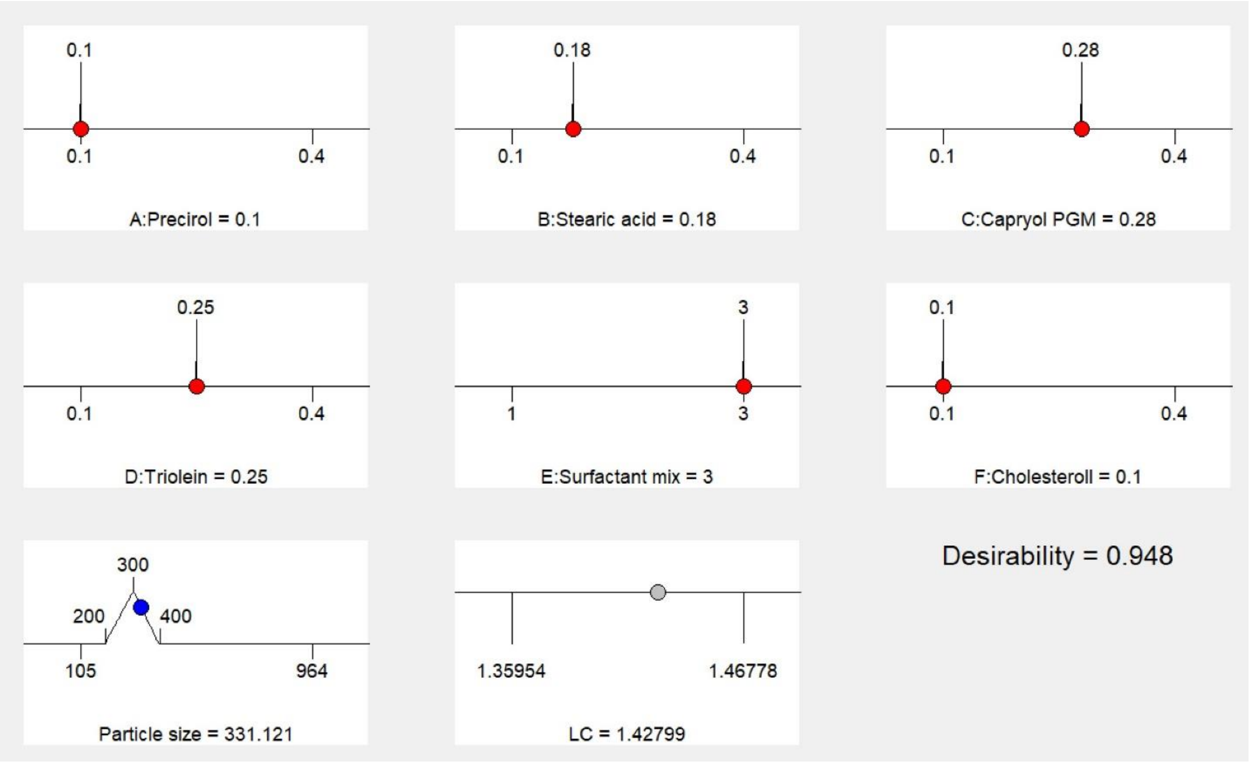

Supplementary Figure S1. Particle size targeting (target size of ~300 nm) of BCT-TRT loaded niosomes through the prediction of various independent variables (A, B, C, D, E, and F) with high desirability of 0.948.

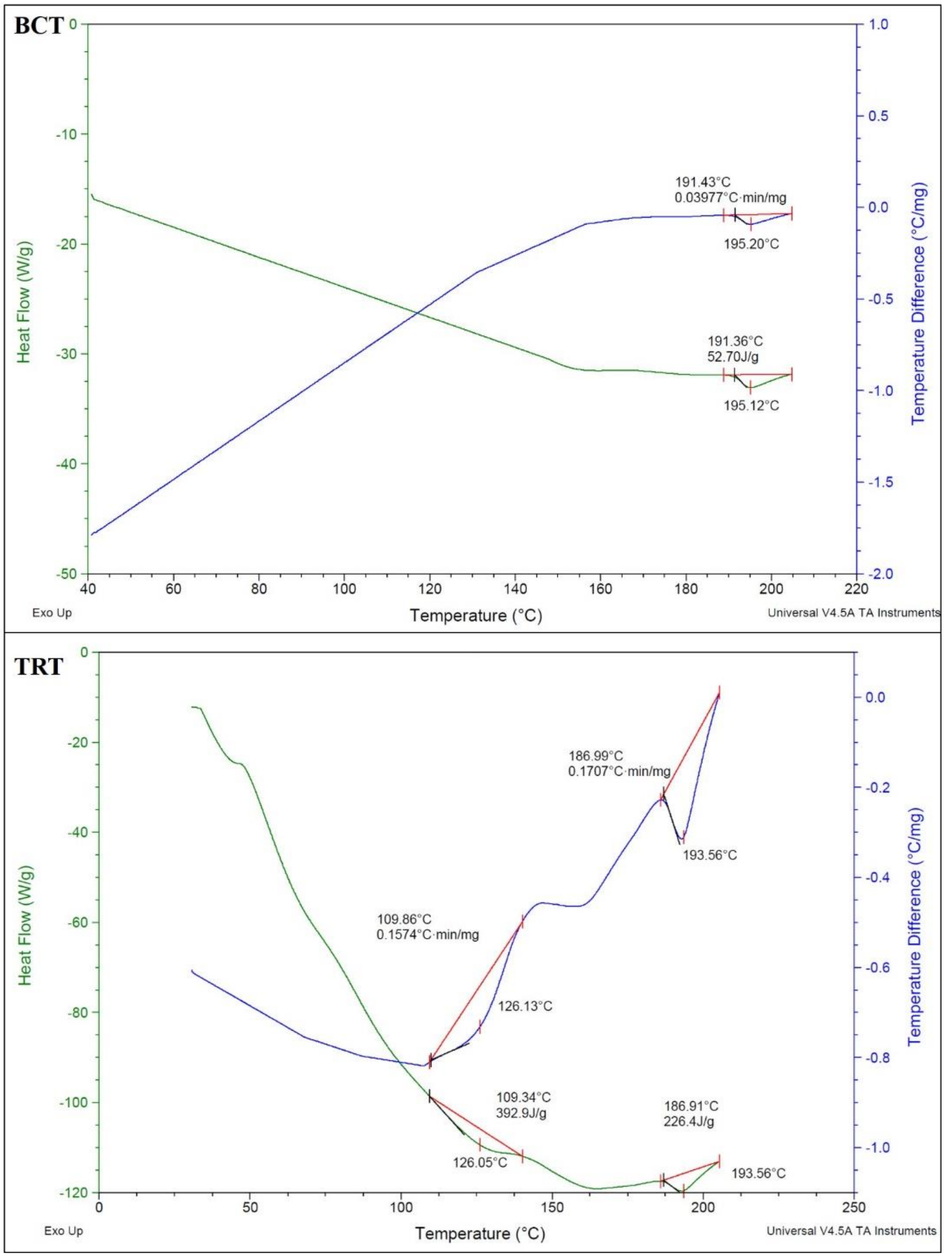

Supplementary Figure S2. Representative DSC thermograms (in blue color) and TGA thermograms (in green color) of bicalutamide (BCT) and tretinoin (TRT)

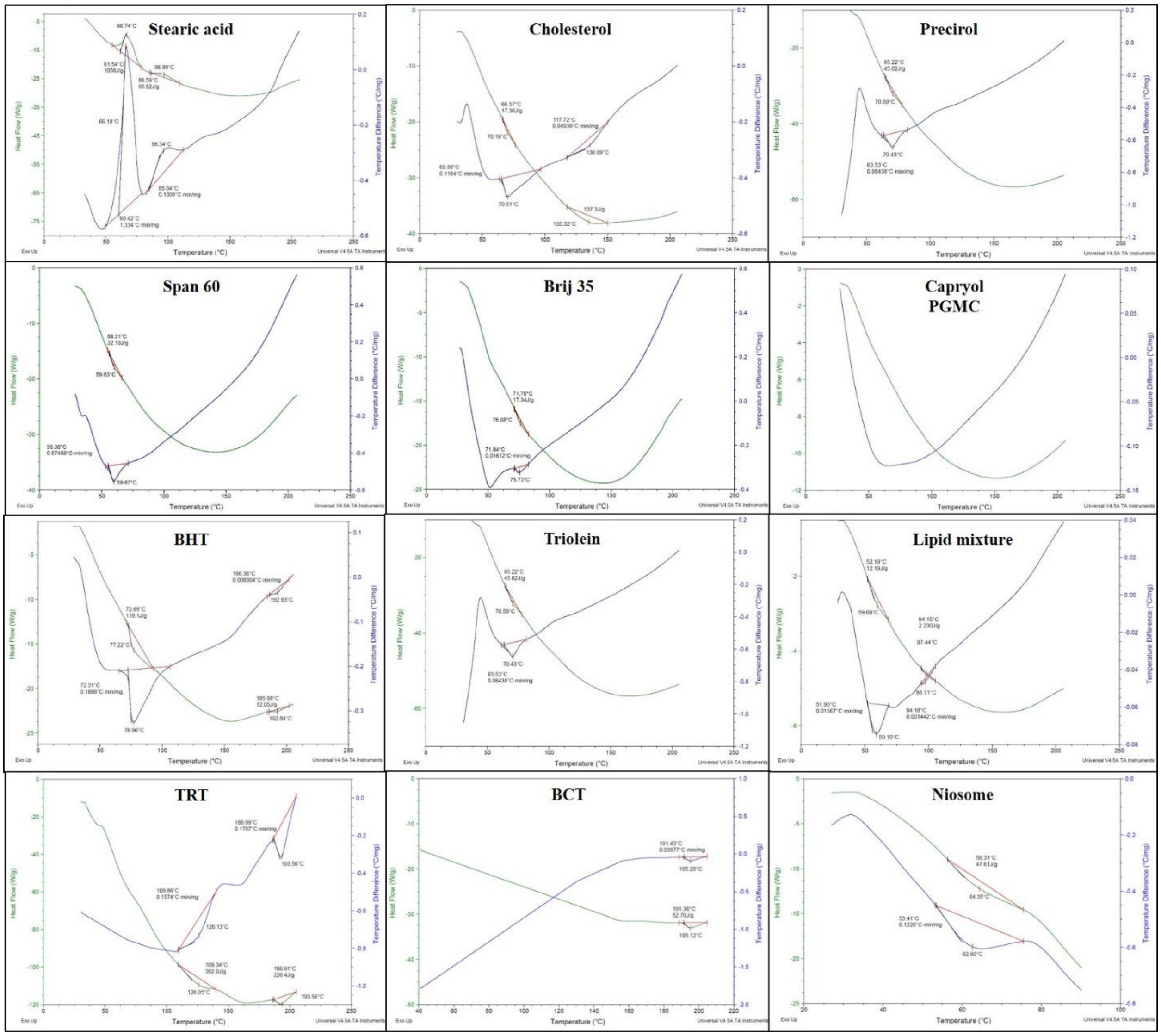

Supplementary Figure S3. Representative DSC thermograms (in blue color) and TGA thermograms (in green color) of stearic acid, cholesterol, Precirol, triolein, Capryol PGMC, Span 60, Brij 35, butyrated hydroxyl toluene (BHT), bicalutamide (BCT), tretinoin (TRT), lipid mixture, and niosome.

ANOVA for Response Surface 2FI model (Response variable: Particle size)						
**Source**	**Sum of Squares**	**df**	**Mean Square**	**F Value**	**p-value Prob > F**	
**Model**	**0.98**	**21**	**0.046**	**2.50**	**0.0329**	**Significant**
A-Precirol	*9.732E-003*	*1*	*9.732E-003*	*0.52*	*0.4795*	
B-Stearic acid	***0.090***	***1***	***0.090***	***4.87***	***0.0423***	***Significant***
C-Capryol PGM	*0.032*	*1*	*0.032*	*1.72*	*0.2078*	
D-Triolein	*5.738E-003*	*1*	*5.738E-003*	*0.31*	*0.5860*	
E-Surfactant mix	*8.410E-004*	*1*	*8.410E-004*	*0.045*	*0.8342*	
F-Cholesteroll	*9.078E-004*	*1*	*9.078E-004*	*0.049*	*0.8278*	
AB	*9.624E-003*	*1*	*9.624E-003*	*0.52*	*0.4819*	
AC	*0.016*	*1*	*0.016*	*0.86*	*0.3665*	
AD	*1.163E-003*	*1*	*1.163E-003*	*0.063*	*0.8056*	
AE	*5.413E-003*	*1*	*5.413E-003*	*0.29*	*0.5967*	
AF	*4.947E-003*	*1*	*4.947E-003*	*0.27*	*0.6128*	
BC	*0.028*	*1*	*0.028*	*1.49*	*0.2393*	
BD	*2.858E-008*	*1*	*2.858E-008*	*1.539E-006*	*0.9990*	
BE	*0.034*	*1*	*0.034*	*1.82*	*0.1967*	
BF	*0.031*	*1*	*0.031*	*1.68*	*0.2138*	
CD	*6.247E-004*	*1*	*6.247E-004*	*0.034*	*0.8568*	
CE	*0.062*	*1*	*0.062*	*3.32*	*0.0871*	
CF	*1.790E-003*	*1*	*1.790E-003*	*0.096*	*0.7602*	
DE	***0.23***	***1***	***0.23***	***12.44***	***0.0028***	***Significant***
DF	***0.11***	***1***	***0.11***	***6.06***	***0.0256***	***Significant***
EF	***0.100***	***1***	***0.100***	***5.38***	***0.0339***	***Significant***
Residual	0.30	16	0.019			
Lack of Fit	***0.27***	***11***	***0.024***	***3.88***	***0.0729***	***Not significant***

Supplementary Table S1. ANOVA results for Response Surface 2FI model (Response variable: Particle size)
